# Jumping Mechanography: Reference Centiles in Childhood and Introduction of the Nerve–Muscle Index to Quantify Motor Efficiency

**DOI:** 10.3390/jcm12185984

**Published:** 2023-09-15

**Authors:** Kyriakos Martakis, Ute Alexy, Christina Stark, Andreas Hahn, Rainer Rawer, Ibrahim Duran, Eckhard Schönau

**Affiliations:** 1 Department of Pediatrics, Faculty of Medicine and University Hospital Cologne, Kerpener Strasse 62, 50937 Cologne, Germany; kyriakos.martakis@uk-koeln.de (K.M.);; 2 Department of Pediatric Neurology, Justus-Liebig-University Giessen, Feulgen Str. 10-12, 35392 Giessen, Germany; 3 Department of Nutritional Epidemiology, Institute of Nutritional and Food Science, University of Bonn, 53115 Bonn, Germany; alexy@uni-bonn.de; 4 Department of Neurology, Faculty of Medicine and University Hospital Cologne, Kerpener Strasse 62, 50937 Cologne, Germany; christina.stark@uk-koeln.de; 5 Novotec Medical GmbH, 75172 Pforzheim, Germany; r.rawer@novotecmedical.de; 6 Center of Prevention and Rehabilitation, UniReha, Faculty of Medicine and University Hospital Cologne, Lindenburger Allee 44, 50931 Cologne, Germany

**Keywords:** Nerve–Muscle Index (NMI), jumping mechanography, single-two-legged jump, DONALD study, force, velocity, power, obesity, motor efficiency

## Abstract

Jumping mechanography provides robust motor function indicators among children. The study aim was to develop centiles for the single 2-leg jump (S2LJ) in German children and adolescents and to identify differences in children with obesity. Data were collected in 2004–2021 through the German DOrtmund Nutritional and Anthropometric Longitudinally Designed (DONALD) study. All participants (6–18 years, mean age 11.4) performed annually an S2LJ aiming for maximum height on a Ground Reaction Force Platform. LMS (lambda-mu-sigma), including resampling, was used to develop centiles for velocity (v_max_), jump height (h_max_), relative force (F_max_/BW), relative power (P_max_/mass), impulse asymmetry and a new parameter to describe jump efficiency, the Nerve–Muscle Index (NMI), defined as v_max_/(F_max_/BW). Data from 882 children and adolescents were analyzed (3062 measurements, median 3 per individual). In females, F_max_/BW values were higher in younger age but remained constant in adolescence. v_max_, h_max_ and P_max_/mass increased in childhood, reaching a plateau in adolescence. In males, v_max_, h_max_ and P_max_/mass showed a constant increase and the F_max_/BW remained lower. Children with obesity showed lower F_max_/BW, h_max_, v_max_ and the NMI, hence, lower velocity per relative force unit and less efficient jump. The centiles should be used to monitor motor development in childhood. The NMI is a surrogate for motor efficiency.

## 1. Introduction

Evaluation of muscle function is crucial in childhood, e.g., to understand the muscle–bone relationship and/or monitor the motor development of the child [1,2]. Muscle function assessment can be performed using isometric tests among others, such as hand-held dynamometry, isokinetic and field tests, but also using dynamic, functional tests, such as jumping mechanography [3].

Jumping mechanography is valid and reliable to measure ground reaction forces associated with a standardized jump in children [4,5]. Such a standardized jump is the single 2-leg jump (S2LJ) performed on a Ground Reaction Force Plate (GRFP) [5]. Relative maximum force (F_max_/Bodyweight), relative power (P_max_/mass), speed and maximum jump height (h_max_) are common parameters assessed during the measurement. Thus, this test could allow a rather simple examination of various domains of physical fitness, such as strength, power or coordination, in a differentiated manner.

In the previous literature, there have also been some attempts to quantify the efficiency of the jump by combining individual measurement parameters (e.g., “force efficiency”, defined as $\frac{EFI}{\frac{Fmax}{BW}\times \frac{1}{2.4}}$, where *EFI* was defined as $\frac{Pmax}{mass}\;$, expressed as percentage of the expected age- and gender-adjusted value). A jump was classified as efficient if it achieves a high level of performance with as little force as possible. Age-related changes and sex-associated differences in the jumping pattern are expected in childhood and adolescence [6]. Regardless of sex, muscle size changes and maturation of neuromuscular function can significantly influence strength gains during growth. These processes are expected to differ in males and females [7].

Thus, reference values, which in childhood should also be age-related and sex-specific are necessary to evaluate these results [5,8]. Pediatric reference centiles are available for the S2LJ among healthy children from Canada (mean (SD): age 13.6 (3.0) years) and the Czech Republic (mean (SD): age 11.8 (3.5) years) [4,9]. However, although S2LJ reference data for German children have are already been published (range 3–19 years) [10], age-related centiles are actually not available. Finally, there are insufficient data regarding motor development in children with increased bodyweight. However, experience from cross-sectional data has shown that children and adolescents with obesity show increased force and relative power for the S2LJ, while variances in the structure and function of the muscles and bones of the legs are also expected [1,11].

Hence, the aims of this study were: (i) developing of age-dependent centile curves for the kinetic parameters of an S2LJ among healthy boys and girls from the 6th to the 18th year of age in Germany using data from the open cohort DOrtmund Nutritional and Anthropometric Longitudinally Designed (DONALD) study; (ii) introducing a new mechanography-related parameter to quantify jump efficiency; (iii) evaluating jumping performance during childhood development; and (iv) identifying differences in the jump characteristics of children with obesity, implementing the presented centiles and parameters, hypothesizing a probably differently coordinated jump in children with obesity. Ideally, the presented tools can help monitor motor development in children.

## 2. Materials and Methods

### 2.1. Study Design

Every year since 1985, a small group of infants from the metropolitan Dortmund area are recruited in the open cohort DONALD study. These children participate in observational study visits throughout infancy, childhood and adolescence, until adulthood. Within the framework of the DONALD study, detailed data on nutritional intake, growth, development, metabolism and health status are collected at regular, usually annual, intervals. These data can be used to examine the diet–health or growth–health relationships, but also to create reference data from healthy children and adolescents [12]. The state of North Rhine-Westphalia grants basic funding to the DONALD study. Since 2012, the study is affiliated with the Department of Nutritional Epidemiology of the Institute for Nutrition and Food Sciences (IEL) of the Rheinische Friedrich-Wilhelms University of Bonn. The principles of the Declaration of Helsinki (2013), as well as the Declaration of Taipei on Ethical Considerations regarding Health Databases and Biobanks (2016), have been respected. The study is registered in the German Clinical Trial Register (DRKS00029092).

### 2.2. Anthropometrics

Anthropometric data were collected at all visits at the study center. Body height has been recorded with an approximation of 0.1 cm using a digital telescopic wall-mounted stadiometer (Harpenden, Rappenswil, Switzerland). Body weight has been measured with an electronic scale (Model 753 E; Seca, Hamburg, Germany) at an approximation of 0.1 kg [12]. We used the definitions of underweight, normal weight, overweight and obesity, as well as the BMI Z-Scores reference system of Kromeyer-Hauschild [13].

### 2.3. Mechanography

The assessment of the participants’ physical activity and performance has always been included in the study items. Since June 2004, jumping mechanography has been included biannually in the study visits, from the sixth year of age [14]. For the assessment, we used a GRFP (Leonardo Mechanograph^®^, NOVOTEC Medical GmbH, Pforzheim, Germany). According to the study protocol, the children, only wearing underwear and no shoes, performed a single two-leg jump (S2LJ), aiming for maximum height. The children stood on the GRFP with each foot placed on one section of the GRFP. The GRFP consists of two individual smaller platforms, which have the capacity to record vertical force separately for the left and right leg. The counter-movement jump was performed allowing free movement of arms and without any other restrictions. The children were instructed to jump as high as possible using both feet and landing on both feet [5].

To evaluate the jump efficiency, we introduced the Nerve–Muscle Index (NMI), defined as the ratio of maximum velocity to relative force, hence v_max_/(F_max_/Bodyweight). Since power is velocity multiplied by force, we found it useful to interrelate the two factors and assess whether the observed performance was achieved more by the velocity-factor (surrogate for neuro-motor coordination) or by the force-factor (surrogate for muscular strength).

For the analyses, we used R Studio (Version 2022.07.2 Build 576), R version 4.2.1 and the gamlss R-package, version 5.4–12. Descriptive statistics included primarily auxological and jump parameters, as well as demographics. Age-related centiles were calculated for the variables maximal velocity (v_max_, in m/s), maximal jump height (h_max_, in m), maximal power in relation to mass (P_max_/mass, in W/kg), maximal force related to bodyweight (F_max_/BW, in N/N) and the Nerve–Muscle Index (NMI), as well as asymmetry of the jump impulse (product of mass and velocity) during landing (difference in the impulse, or DIMP, in %) stratified by sex.

### 2.4. Statistical Treatment of Data

For the development of the centile curves for S2LJ v_max_, we initially used the classic LMS (lambda-mu-sigma) method [15,16]. The ZLMS-scores were calculated using a modified Box-Cox transformation:

(1) $$Z_{LMS}=\frac{1}{S\left(a\right)\times L\left(a\right)}\times \left[{\left(\frac{g}{M\left(a\right)}\right)}^{L\left(a\right)}-1\right]\;\mathrm{for}\;S(a),\;L(a)\;\mathrm{and}\;M(a)\;\neq \;0$$

(2) $$\;Z_{LMS}=\frac{\ln\left(\frac{g}{M\left(a\right)}\right)}{S\left(a\right)}\mathrm{for}\;L(a)=0\;\mathrm{and}\;S(a),\;M(a)\;\neq \;0\;$$

**Figure 1:**
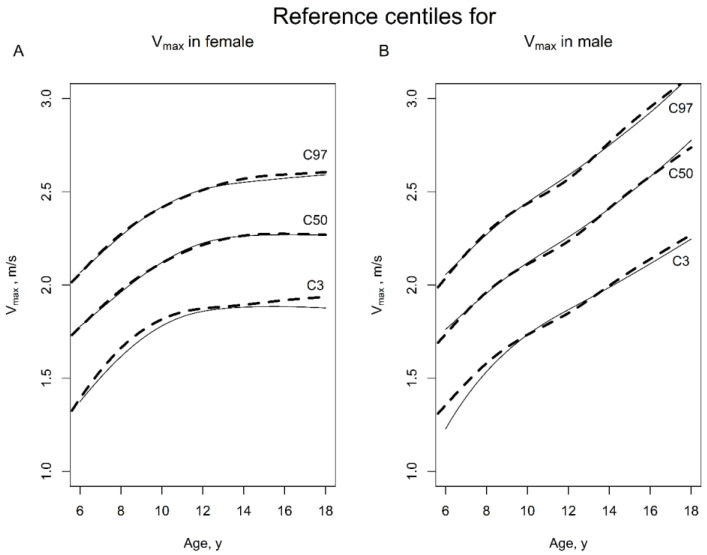
Reference centiles for v_max_ in female and male children, using the LMS method (intermittent line) and the combination of LMS and resampling approach (continuous line) as suggested by Vogel et al. [17], in healthy females (**A**) and males (**B**) in Germany, in the age range of 6–18 years.

The methodology for the calculation of Z-scores, adjustment of skewness *L*(*a*), median value *M*(*a*) and the coefficient of variation *S*(*a*) were adjusted to the data with the maximum likelihood estimate, applying cubic spline interpolation. The centile estimations were performed with the R package GAMLSS and the function gamlss. We used the BCCG distribution to estimate *M*(*a*), *S*(*a*) and *L*(*a*). These age-related centile curves are presented in Figure 1 with an intermittent line.

However, it is known that data selections from cohort studies among healthy children often include biases, as shown in the Life Child study of the Leipzig Center for Civilization Diseases [17]. Biases such as the data collection among members of the same family or subjects who may contribute with different numbers of measurements, but also subjects having been assessed approximately around the chronological annual birthday of each subject—which was also the case in the present study—can violate the independence assumption for subjects and assessments. The exclusion of all dependent measurements could be an option, which would result, however, in a significant loss of data [17].

To overcome this methodological challenge, Vogel et al. (2017) proposed a new approach, using unbalanced longitudinal data, combining the LMS-type approach and resampling, hence repeating the estimation process 1000 times, using different subsamples [17]. Vogel et al. (2017) suggested that, each time, 75% of the families should be sampled, and in a second step, one measurement from all available measurements of members of each family should be sampled. Additionally, it should be assured that each measurement has the same probability to be selected, using sampling weights on families, but also ensuring that families comprising only one subject would not be over-represented in the resampling process. The method, which was called LMS-P by the authors, has been explained in detail in the respective original publication [17].

Hence, we additionally calculated the age-related centile curves for v_max_ using the LMS-P method and presented them in Figure 1 with a continuous line. The presented differences between the LMS and the LMS-P approach are little but recognizable for children showing lower v_max_ in the S2LJ, both for girls and boys of all ages, as well as for older boys and girls with higher v_max_ values in the S2LJ. This result is consistent with the results described in the literature; that if the number of subjects is large and the number of measurements (per subject) is small, it is possible to ignore the longitudinal aspect of the data and treat it as cross-sectional. This was carried out, e.g., by the World Health Organization for the WHO Child Growth Standards (2009) (https://apps.who.int/iris/bitstream/handle/10665/44026/9789241547635_eng.pdf, accessed on 12 February 2023). Although there were not very relevant differences between the two results, we decided to use the LMS-P method, using the childsds package for R, in version 0.8.0 [17], for the further centile calculation in this study.

### 2.5. Stability of Jump Pattern during Childhood and Adolescents

We calculated the correlation of the Z-scores of the P_max_ and the Z-scores for the NMI between different age classes to analyze the stability of these jump parameters over childhood and adolescence. Since the children were examined almost exclusively biannually on their chronological birthday (6th, 8th, 10th until 18th), we formed intersections of measurements performed; for instance, around the 6th and 8th birthdays, around the 6th and the 10th birthdays, around the 6th and the 12 birthdays, etc. We measured the Z-scores for all of these intersections and calculated the correlations [15,16]. With these correlation coefficients, it is possible to calculate Z-scores for the change in jumping mechanography parameters. To simplify the demonstration of the method, we will only refer to the v_max_ of the S2LJ.

According to Cole [15], the difference between two Z-scores

∆Z = Z2 − Z1(3)

Determined at different time points t2 > t1 is a measure for centile-crossing development. For our reference population, the following applies (because Z-scores have a standard normal distribution):

Expected value E[∆Z] = 0

(4) $$\mathrm{standard}\;\mathrm{deviation}\;\sigma (\Delta Z)=\sqrt{2\times \left(1-r\right)}$$

where r is the correlation coefficient between Z2 and Z1. Thus, for the SD of ΔZ values:

(5) $$\mathrm{SD}\;\mathrm{for}\;\mathrm{centile}\;\mathrm{change}=\frac{\Delta Z\;}{\sqrt{(2\times \left(1-r\right)}}$$

Thus, the correlation coefficient r depends on the age of the child and the time interval between two S2LJ measures. With the dataset of n = 1445 measurements performed by girls and 1617 measurements by boys, the correlation coefficients r were calculated for all above defined intersections for the ages between 6 and 18 years. Since the Z-scores have a standard normal distribution, the Z-scores for their change is equal to the effect size of the size and can interpreted similar to Cohen’s d.

### 2.6. Obesity-Associated Changes in the S2LJ Parameters

To analyze the relationship between obesity and the jumping characteristics of healthy children, jump parameters of participants with obesity were compared with their values from the non-obese participants [18]. For the inferential analysis, we used a Wilcoxon test (*p* value significance < 0.05) for non-normally distributed data, and to measure the effect size of the differences we calculated Cohen’s d. Since we compared Z-scores, no further adjustment for age or sex was needed. In addition, mixed effect linear models were used to confirm the results of the above-mentioned analysis, since these models can handle better the hierarchical structure of the data (repeated measurements; some participants belonged to one family).

## 3. Results

### 3.1. Study Population

Study sample characteristics are presented in Table 1. To calculate the centile-curves, 3062 measurements, performed by a total of 882 children from the ages of 6 to 18 years, from 656 different families, were analyzed. Among these measurements, 1445 were performed by girls and 1617 by boys. The mean BMI during the assessments was 18.7 kg/m^2^ for girls and 18.8 kg/m^2^ for boys. To investigate the relationship between increased bodyweight and the S2LJ parameters, we compared the measurements in participants with obesity (BMI ≥ 97th centile) at the time of measurement (15 females and 20 males with 57 measurements) and 847 normal weight children (3005 measurements).

### 3.2. Reference Centiles for S2LJ

**Figure 2:**
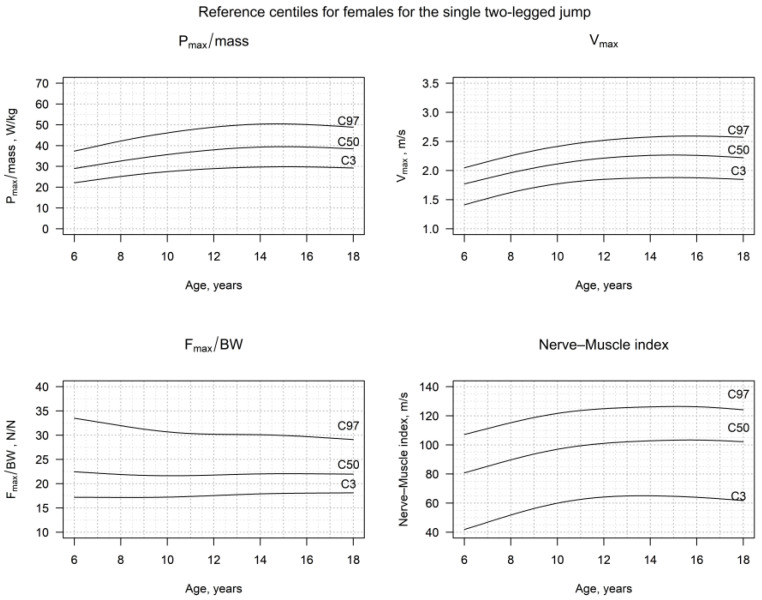
Reference centiles for P_max_/mass, v_max_, F_max_/BW and the Nerve–Muscle Index (NMI) in healthy females in Germany, in the age range of 6–18 years.

**Figure 3:**
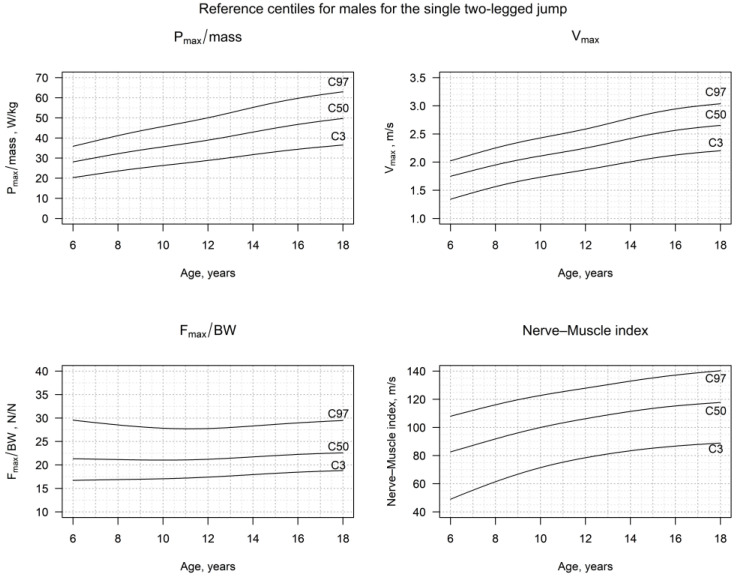
Reference centiles for P_max_/mass, v_max_, F_max_/BW and the Nerve–Muscle Index (NMI) in healthy males in Germany, in the age range of 6–18 years.

The detailed centiles for the v_max_, h_max_, P_max_/mass, F_max_/BW, the NMI and side-difference of the impulse for girls and boys, truncated to three decimal places, are presented in Figure 2 and Figure 3, as well as in Tables S1–S12, and in Figures S1 and S2 in the Supplement. The v_max_, h_max_ and P_max_/mass showed a significant increase in both females and males, until late childhood. This trend continued in males until adulthood, but showed a plateau in females, starting from the 13th year of age. On the other hand, the F_max_/BW, as well as the side-difference, remained very stable, especially after the age of 8 to 9 years for both sexes. Thus, the NMI showed an evolution according to the one of P_max_/mass, showing a continuous increase during childhood and adolescence in boys, but reaching a plateau in adolescent girls.

### 3.3. Stability of Jump Pattern during Childhood and Adolescents

Further, we calculated the correlation coefficient r for the Z-scores of the P_max_/mass and the NMI in the interceptions of measurements performed at different age classes for females and males. We summarized the results in Table 2 and Table 3.

### 3.4. Obesity-Associated Changes in the S2LJ Parameters

We identified 57 S2LJs performed by 35 children (15 females, 20 males) with obesity (BMI Z-score ≥ 1.88). The study of their jump characteristics showed significantly lower Z-scores for F_max_/BW (*p* < 0.001) as well as for h_max_ (*p* < 0.001), P_max_/mass (*p* < 0.001) and v_max_ (*p* < 0.001), in comparison to the S2LJs of their non-obese peers (n = 3005; Table 4) The ratio NFI was also significantly lower in obese children (*p* = 0.001) in comparison to their non-obese peers. The effect size was highest for v_max_ with a Cohen’s d of 1.3 and least for F_max_/BW with a Cohen’s d of 0.51 (Table 4).

**Figure 4:**
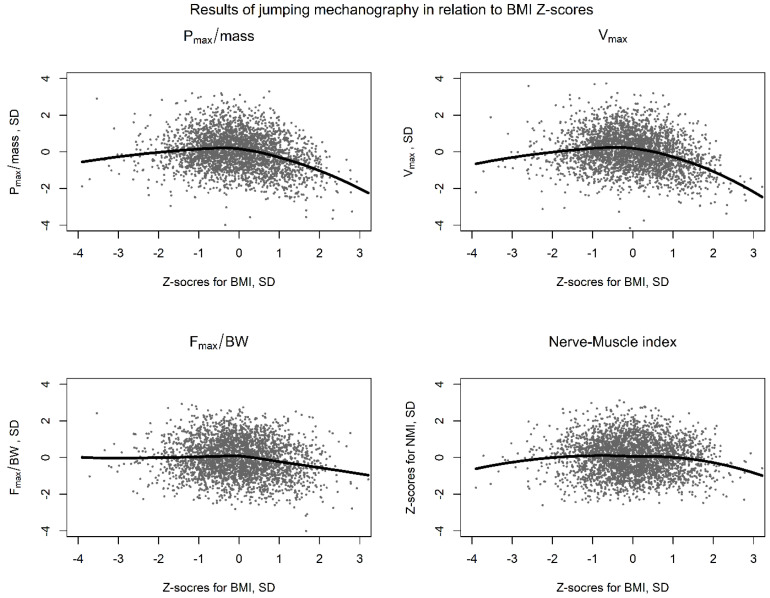
Z-scores for P_max_/mass, v_max_, F_max_/BW and the Nerve–Muscle Index in children in relation to BMI Z-scores in children with obesity in Germany (6–18 years). The solid lines show the results of a Loess (Locally Weighted Scatterplot Smoothing) regression analysis for the four main parameters of the jumping mechanography.

The association of the BMI and the Z-scores for P_max_/mass, v_max_, F_max_/BW and the NMI is also graphically demonstrated, using a non-linear LOESS regression, in Figure 4. Interestingly, the S2LJ performance of children with very low BMI was also not ideal, showed though better v_max_ and P_max_/mass scores, compared to their obese peers.

To confirm our findings, we also performed a mixed effect linear regression to investigate if the Z-scores of BMI are correlated with the Z-scores of the jump parameters. The results were comparable with the aforementioned analysis, with BMI Z-scores significantly negatively correlating with the Z-scores for v_max_ (fixed effects estimate: h_max_; FEE: −0.170, SE: 0.027, *p* < 0.001), P_max_/mass (FEE: −0.225, SE: 0.027, *p* < 0.001), F_max_/BW (FEE: −0.124, SE: 0.026, *p* < 0.001) and the NMI (FEE: −0.058, SE: 0.024, *p* = 0.018) and showing no correlations with the DIMP Z-scores (FEE: −0.008, SE: 0.024, *p* = 0.744).

## 4. Discussion

In the current study, we presented the first sex-specific and age-related reference centiles based on longitudinal data for jumping mechanography outcomes in German children and adolescents from 6 to 18 years of age (using the S2LJ with free swinging arms). Our findings complement previously published data of cross-sectional studies for the S2LJ assessment, but also other jumping assessments in healthy children [4,9,10,19].

### 4.1. Reference Centiles for S2LJ

Our data showed, both in females and males, lower values for h_max_, v_max_ and P_max_/mass, as well as for the newly defined Nerve–Muscle Index (NMI) for younger children, in comparison to those in adolescence. In females, h_max_, v_max_, P_max_/mass and the NMI reached a plateau in adolescence, around the 15th year. In males, the increase was steeper, and the plateau was reached around the 18th year. The F_max_/BW was higher in females during childhood than in males but declined with age, faster during childhood and slower during adolescence. In males, the F_max_/BW was, in general, lower than that of females of the same age and remained constant throughout childhood and puberty. Thus, boys reached the same S2LJ results (h_max_, P_max_/mass) using less force, following a similar pattern to that which has been shown in previous studies [4,9,10]. The observed plateaus in females also correspond with puberty and the age of peak-height velocity, which occur rather early in females [7]. Males did not show such a clear plateau in P_max_/mass, v_max_ and the NMI, though.

The process we recommend to evaluate the S2LJ of an individual child would be as follows:

Step 1: Compare the child’s P_max_/mass with the reference population.

Step 2: Whether the child’s performance is influenced primarily by differences in the v_max_ (parameter depicting neuromuscular coordination), by the F_max_ (muscular strength) or by both.

Step 3: Evaluate the jump efficiency by using the NMI.

Step 4: In special cases, such as in children with unilateral paresis, unilateral spasticity, jump symmetry and, less probably, h_max_ may also be evaluated, using the curves presented in the Supplement File.

Our results are consistent with those proposed by other cross-sectional studies, which also show similar differences between the two sexes in h_max_, P_max_/mass, F_max_/BW and v_max_ [4,9,10,19]. The role of androgens is, of course, crucial for the muscle mass, the prospective height that a child will reach [14] and the bone maturity, as being a factor that differs significantly both in a quantitative as well as in a temporal manner, in male and female adolescents [20,21,22]. Thus, it is obvious that sex-specific centiles should be used to monitor functional changes in the S2LJ parameters in children and adolescents. Future studies should examine if muscle mass differences, training and eating habits, or other factors, play a significant role in the different evolution of S2LJ parameters, not only between boys and girls, but also among individuals of the same sex, especially in adolescence.

### 4.2. Stability of Jump Pattern during Childhood and Adolescents

In the following, we have presented a detailed implementation of the tool to calculate changes in the Z-scores for the P_max_/mass, as summarized in Table 2. We calculated changes in the NMI accordingly and presented the Z-scores in Table 3.

Let us assume a boy who had a Z-score for P_max_/mass of 0.1 at the age of 10 years and Z-score of −0.5 at 16 years. The question is if this deterioration is relevant or a frequent age-related fluctuation, as seen in the reference population. For the P_max_ of the boy we are studying, Formula (5) can be transformed as follows:

(6) $$\mathrm{SD}\;\mathrm{for}\;\mathrm{centile}\;\mathrm{change}=\frac{\left(-0.5\right)-0.1\;}{\sqrt{(2\times \left(1-0.66\right)}}=\frac{-0.6\;}{0.82}=-0.73$$

Thus, the boy showed a deterioration in these six years. The aforementioned calculated value also represents the effect size for the change for this boy between 10 and 16 years, which is moderate, according to Cohen (0.5 to 0.8) [23]. We would then summarize the development as a moderate deterioration. Thus, the correlation coefficient signalizes whether changes over time in the S2LJ parameters of an individual would occur more or less frequently, in comparison with the reference population.

### 4.3. Nerve–Muscle Index and Obesity-Associated Jump Pattern

In this study, we introduced the Nerve–Muscle Index. We chose to use the term “Nerve” referring to the phenomenon of coordinated interaction of muscles, while the term “Muscle” refers to pure muscular strength, and “Index” hints that the quotient of these two parameters can be used as an efficiency parameter for jumping, in this case, but probably also to quantify motor efficiency in general.

The meaning of this parameter is clearer, when we study the S2LJ pattern in a subpopulation of our cohort, such as the group of physically healthy, but children with obesity (BMI Z-score ≥ 1.88) [18]. The study of the children and adolescents with obesity revealed that they showed that the relative parameters (P/mass and F_max_/BW) were significantly decreased, which is a finding that is consistent with already-published cross-sectional data [1]. The children also showed a lower v_max_ in comparison with their non-obese peers. Thus, these children shower higher F_max_/BW in relation to the reached v_max_ (which leads to a reduction in the NMI), indicating that the movement concept has changed. The reason might be associated with lower movement efficacy due to lack of muscle coordination, lower flexibility and/or poorer ability to store energy (stretch-shortening cycle) [11]. Hence, in children with obesity, P_max_/mass is reduced and F_max_/BW seems to be proportionally less reduced than v_max_. The NMI was also significantly lower in children with increased weight, indicating that the lower number of v_max_ units are developed per F_max_/BW unit.

Achieving the highest power with the smallest amount of force, while storing—and saving—the maximum energy is ideal for locomotion coordination and efficiency [24]. Obese children showed lower Z-scores for P_max_/mass and NMI values and, thus, a less efficient jump. The jump pattern of children in children with cystic fibrosis, as well as young adults with congenital heart disease, showed also increased absolute forces and relative low power [25,26]. The study of the NMI in such cohorts could also reveal differences in the performance, for instance of children with heart conditions who are also overweight, in comparison to their non-obese peers.

### 4.4. Limitations

Our study cohort presents a convenience sample of boys and girls from the metropolitan area of Dortmund, which probably may not qualify to represent the totality of German children. To address selection bias and the dependent character of the data, we used the LMS method combined with resampling [17]. The assessments have been performed by a small pool of trained raters, increasing the precision and the reliability of the test.

In our cohort, we found a lower Esslinger Fitness Index (EFI) (Table 1) in comparison to the one of Busche et al., with a comparable scatter, while girls showed a much smaller variation than the boys [10]. Although the physical performance of children may also have shown a significant decrease over one decade in another study in Germany, we found no relevant age-dependence (r = −0.1, test date (measured as time distance from the earliest measurement) vs. EFI) in our cohort [27]. Thus, differences should probably be attributed to selection bias.

Further, a variant for the execution of the S2LJ has been proposed, arguing that the hands-on-waist method is associated with a less variable jump performance due to the effects of the upward swing of arms, as well as with a lower jump height [28]. This variant has been used by Gabel et al. to publish reference centiles for healthy children in Canada, arguing that, because of increased reproducibility, the method is most suitable for field studies [9]. On the contrary, our cohort showed actually lower h_max_ values in both males and females in comparison to theirs [9]. No conclusions regarding reliability can be drawn from our data. Since the sample of children with obesity was rather small, the depicted differences should be studied further in larger cohorts of children with obesity.

## 5. Conclusions

The presented sex-specific, age-related reference centiles for the single two-leg jump of healthy boys and girls aged 6–18 years provide a valid data basis for assessing jump performance in German children and adolescents (and probably comparable populations). The presented centiles for P_max_/mass and the NMI can be also used to monitor motor development and efficiency of children and could be used to counsel for promotion of physical activity, not only in healthy children and adolescents, but also in those suffering from chronic conditions. Children with obesity showed lower jump efficiency and performance at the S2LJ when compared to their non-obese peers. The ratio of the Nerve–Muscle Index, which was introduced and validated in the present study, could be used as a surrogate for jump efficiency, as well as other types of motor efficiency, giving new insights into the evaluation of motor functions and differentiation between the relevant components of muscle force and jump coordination.

## Figures and Tables

**Table 1:** Sample characteristics.

	Females	Males	Total
Participants, n	431	461	882
Measurements, n	1445	1617	3062
Measurements per individual, median (range)	3 (1–7)	3 (1–7)	3 (1–7)
Age, years	11.4 (3.9)	11.4 (3.8)	11.4 (3.9)
Body height, cm	148.2 (19.3)	151.7 (23.0)	150.0 (21.4)
Height, Z-Score	0.37 (0.95)	0.36 (1.02)	0.36 (0.99)
Body mass index (BMI), kg/m^2^	18.7 (3.5)	18.8 (4.0)	18.8 (3.8)
BMI, Z-Score ^1^	−0.05 (0.88)	−0.03 (0.96)	−0.04 (0.92)
Esslinger Fitness Index (EFI), Z-Score	−0.57 (0.82)	−0.67 (1.07)	−0.62 (0.96)
Measurement without obesity, n	1427	1589	3005
Measurements with obesity, n	19	38	57

^1^ Z-scores were calculated with the reference centiles of the KIGGS study (ISBN 978-3-89606-218-5). All values are mean (SD), if not otherwise stated.

**Table 2:** Correlation of the Z-scores for relative P_max_ and age in years in healthy males and females in Germany, in the age range of 6–18 years.

	Z-Scores for Rel. P_max_ in Males, (95%. CI Values)						
Age (Years)	6	8	10	12	14	16	18
6		0.66 (0.56; 0.75) n = 134	0.52 (0.39; 0.64) n = 128	0.60 (0.46; 0.71) n = 103	0.42 (0.21; 0.59) n = 74	0.42 (0.17; 0.62) n = 53	0.53 (0.22; 0.74) n = 33
8	0.60 (0.48; 0.70) n = 132		0.67 (0.57; 0.75) n = 140	0.65 (0.53; 0.74) n = 113	0.54 (0.37; 0.67) n = 93	0.57 (0.36; 0.72) n = 57	0.63 (0.41; 0.78) n = 44
10	0.47 (0.31; 0.61) n = 100	0.64 (0.52; 0.74) n = 117		0.68 (0.58; 0.76) n = 145	0.61 (0.48; 0.71) n = 116	0.66 (0.51; 0.76) n = 83	0.63 (0.45; 0.76) n = 61
12	0.48 (0.29, 0.64) n = 79	0.59 (0.44; 0.70) n = 98	0.55 (0.39; 0.67) n = 101		0.60 (0.47; 0.70) n = 126	0.66 (0.52; 0.76) n = 94	0.64 (0.47; 0.76) n = 68
14	0.51 (0.31; 0.67) n= 66	0.55 (0.38; 0.69) n = 82	0.57 (0.41; 0.70) n = 89	0.72 (0.61; 0.80) n = 111		0.74 (0.64; 0.82) n = 104	0.51 (0.32; 0.65) n = 79
16	0.39 (0.09; 0.62) n = 42	0.45 (0.19; 0.65) n = 48	0.51 (0.29; 0.68) n = 59	0.61 (0.44; 0.73) n = 74	0.70 (0.58; 0.79) n = 94		0.80 (0.72; 0.87) n = 94
18	0.35 (−0.02; 0.63) n = 29	0.43 (0.17; 0.64) n = 46	0.31 (0.03; 0.55) n = 48	0.46 (0.25; 0.64) n = 62	0.61 (0.45; 0.73) n = 61	0.73 (0.61; 0.82) n = 79	
Age, years	6	8	10	12	14	16	18

Z-Scores for rel. P_max_ in females.

**Table 3:** Correlation of the Z-scores for the NMI and age in years in healthy males and females in Germany, in the age range of 6–18 years (95%. CI values).

	Z-Score for NMI for Males						
Age, Years	6	8	10	12	14	16	18
6		0.28 0.04; 0.36) n = 134	0.14 (−0.03; 0.31) n = 128	0.09 (−0.10; 0.28) n = 103	0.38 (0.16; 0.56) n = 74	0.26 (−0.01; 0.49) n = 53	−0.15 (−0.47; 0.21) n = 33
8	0.35 (0.19; 0.49) n = 132		0.17 (0.01; 0.33) n = 140	0.25 (0.07; 0.42) n = 113	0.22 (0.02; 0.41) n = 93	0.45 (0.22; 0.63) n = 57	0.16 (−0.14; 0.44) n = 44
10	0.19 (0.0; 0.38) n = 100	0.25 (0.07; 0.41) n = 117		0.34 (0.190.48) n = 145	0.34 (0.17; 0.49) n = 116	0.33 (0.13; 0.51) n = 83	0.40 (0.16; 0.59) n = 61
12	0.15 (−0.6; 0.37) n = 79	0.29 (0.10; 0.46) n = 98	0.35 (0.17; 0.51) n = 101		0.31 (0.15; 0.46) n = 126	0.44 (0.26; 0.59) n = 94	0.20 (−0.04; 0.42) n = 68
14	0.26 (0.02; 0.47) n = 66	0.24 (0.02; 0.43) n = 82	0.31 (0.11; 0.49) n = 89	0.52 (0.37; 0.64) n = 111		0.40 (0.22; 0.55) n = 102	0.41 (0.21; 0.59) n = 79
16	0.14 (−0.17; 0.42) n = 42	0.34 (0.06; 0.57) n = 48	0.30 (0.05; 0.52) n = 59	0.60 (0.43; 0.73) n = 74	0.49 (0.32; 0.63) n = 94		0.40 (0.22; 0.56) n = 94
18	0.21 (−0.17; 0.53) n = 29	0.38 (0.10; 0.60) n = 46	0.41 (0.14; 0.62) n = 48	0.51 (0.30; 0.67) n = 62	0.51 (0.32; 0.66) n = 76	0.64 (0.49; 0.76) n = 79	
Age, years	6	8	10	12	14	16	18

Z-score for NMI for females.

**Table 4:** Differences in the S2LJ jumping pattern in children without and with obesity, in males and females in Germany, in the age range of 6–18 years.

	Measurements in Children with Z-Scores for BMI			
Z-Scores for	<1.88 n = 3005	≥1.88 n = 57	*p*-Value	Cohen’s d
v_max_	0.04 (0.01; 0.08)	−1.23 (−1.46; −1.00)	< 0.001	1.30 (1.03; 1.57)
h_max_	0.03 (−0.01; 0.07)	−1.09 (−1.33; −0.84)	< 0.001	1.13 (0.87; 1.40)
P_max_/mass	0.01 (−0.02; 0.05)	−1.13 (−1.38; −0.90)	< 0.001	1.17 (0.91; 1.43)
F_max_/BW	−0.03 (−0.07; 0.01)	−0.54 (−0.82; −0.25)	< 0.001	0.51 (0.25; 0.77)
NMI	0.06 (0.02; 0.09)	−0.38 (−0.63; −0.13)	0.001	0.45 (0.18; 0.71)
DIMP	−0.02 (−0.05; 0.02)	0.20 (−0.06; 0.45)	0.105	na

Data presented as Z-score (CI 95%), or d (CI 95%), BMI: body mass index, v_max_: max velocity, h_max_: max jump height, P_max_: max power, F_max_: max Force, BW: Bodyweight, NMI: nerve–muscle index, DIMP: differences in jump impulse, na: not applicable.

## Data Availability

Data is available upon request to alexy@uni-bonn.de.

## Supplementary Materials

The following supporting information can be downloaded at: https://www.mdpi.com/article/10.3390/jcm12185984/s1, Figure S1. Reference centiles for h_max_ and the side-difference of the impulse in healthy females in Germany, in the age range of 6–18 years, Figure S2. Reference centiles for h_max_ and the side-difference of the impulse in healthy males in Germany, in the age range of 6–18 years, Table S1. Reference centiles for v_max_ in the S2LJ in healthy females in Germany, in the age range of 6–18 years, Table S2. Reference centiles for v_max_ in the S2LJ in healthy males in Germany, in the age range of 6–18 years, Table S3. Reference centiles for h_max_ in the S2LJ in healthy females in Germany, in the age range of 6–18 years, Table S4. Reference centiles for h_max_ in the S2LJ in healthy males in Germany, in the age range of 6–18 years, Table S5. Reference centiles for P_max_/mass in the S2LJ in healthy females in Germany, in the age range of 6–18 years, Table S6. Reference centiles for P_max_/mass in the S2LJ in healthy males in Germany, in the age range of 6–18 years, Table S7. Reference centiles for F_max_/BW in the S2LJ in healthy females in Germany, in the age range of 6–18 years, Table S8. Reference centiles for F_max_/BW in the S2LJ in healthy males in Germany, in the age range of 6–18 years, Table S9. Reference centiles for the Nerve–Muscle Index in the S2LJ in healthy females in Germany, in the age range of 6–18 years, Table S10. Reference centiles for the Nerve–Muscle Index in the S2LJ in healthy males in Germany, in the age range of 6–18 years, Table S11. Reference centiles for the side-difference of the impulse in the S2LJ in healthy females in Germany, in the age range of 6–18 years, Table S12. Reference centiles for the side-difference of the impulse in the S2LJ in healthy males in Germany, in the age range of 6–18 years.
