# Supplementary material for: Jumping Mechanography: Reference Centiles in Childhood and Introduction of the Nerve–Muscle Index to Quantify Motor Efficiency
Source: J Clin Med. 2023 Sep 15;12(18):5984. doi: 10.3390/jcm12185984 (PMC10531761; doi:10.3390/jcm12185984)
Supplement: Supplementary file 1 [file jcm-12-05984-s001.zip › jcm-2525995-supplementary.pdf]

Supplement

Supplement Figures

Supplement Figure S1. Reference centiles for $h_{ma}x$ and the side-difference of the impulse in healthy females in Germany, in the age of 6-18 years.

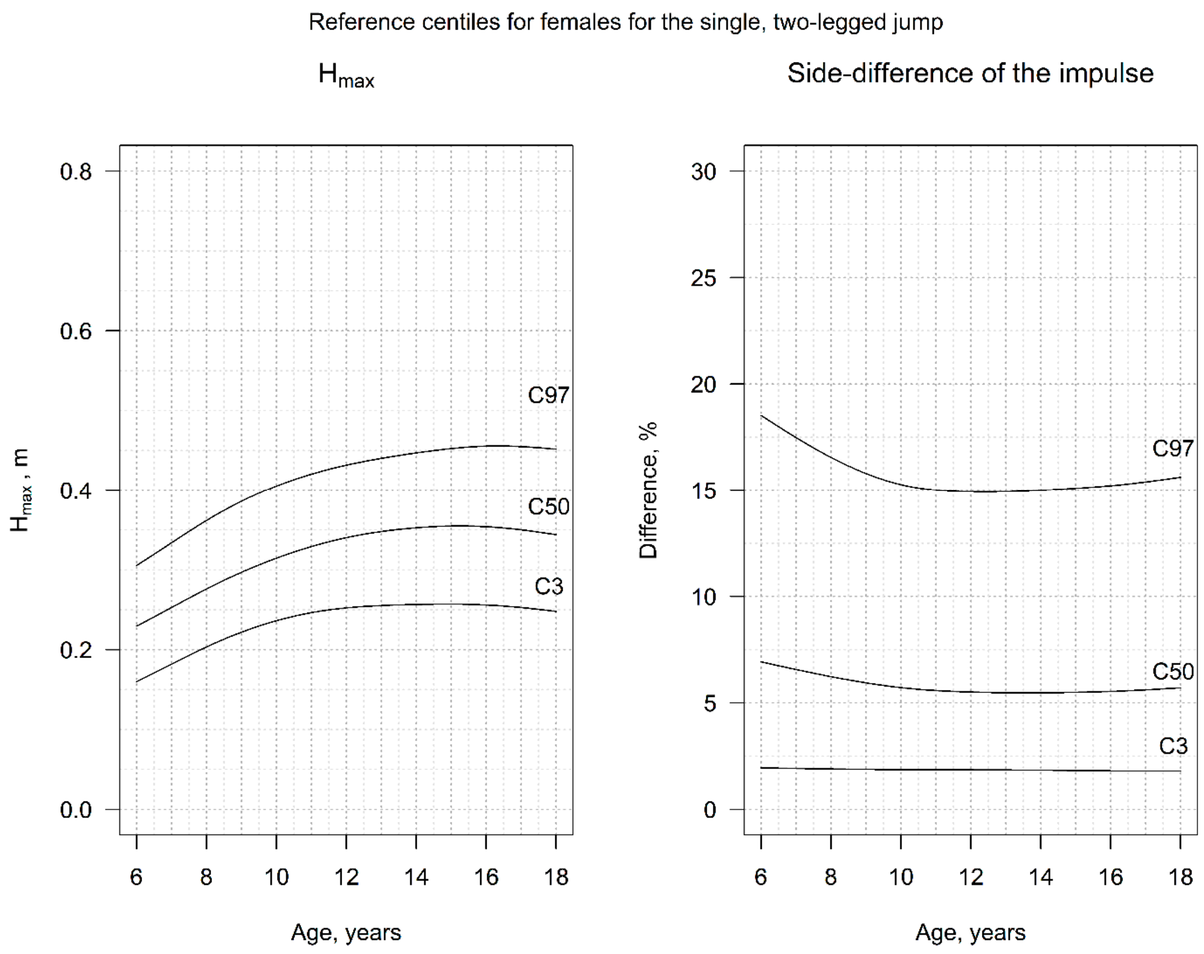

Supplement Figure S2. Reference centiles for h_{max} and the side-difference of the impulse in healthy males in Germany, in the age of 6-18 years.

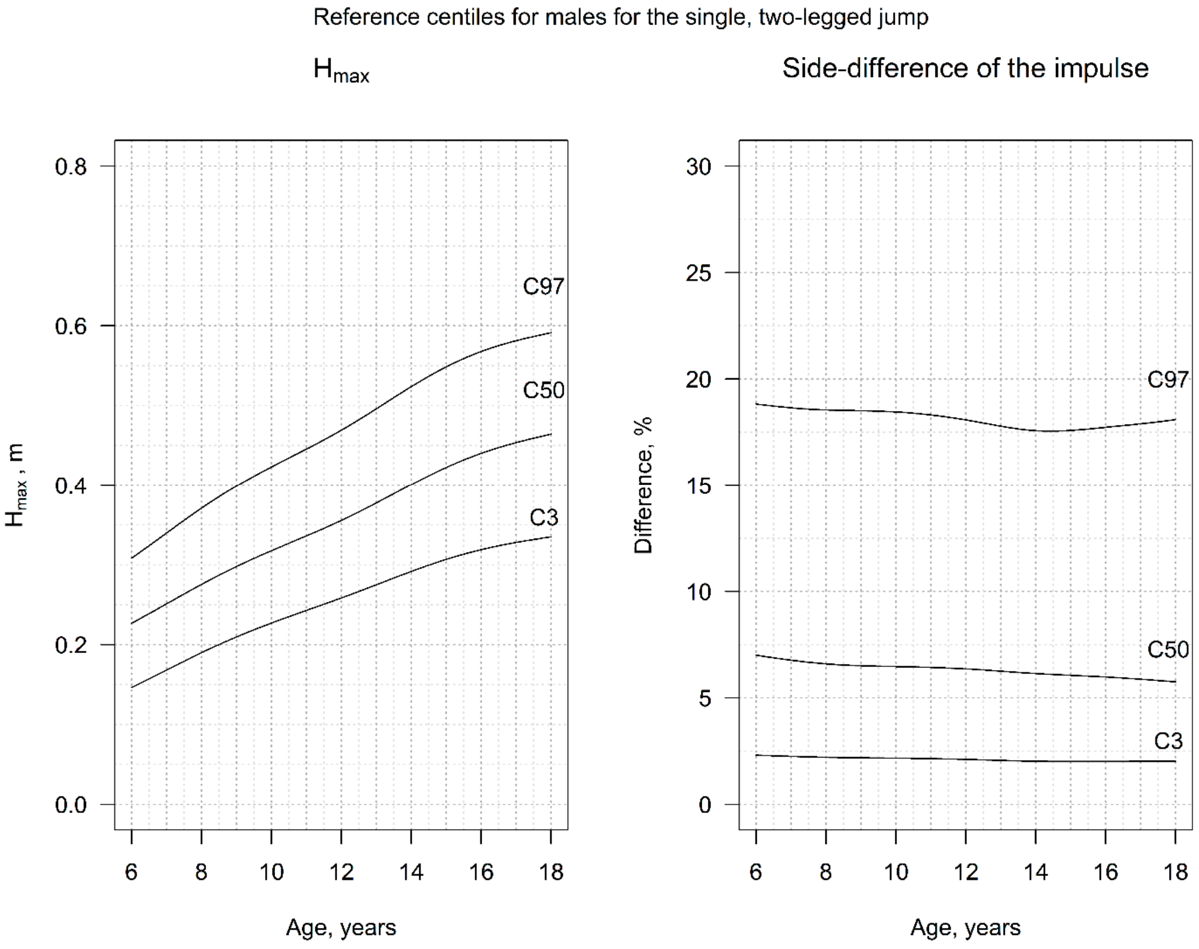

Supplement Tables:

Table S1. Reference centiles for V_{max} in the S2LJ in healthy females in Germany, in the age of 6-18 years.

age	mu	sigma	lambda	C3	C10	C25	C75	C90	C97
6	1,77107109	0,09260358	2,43074625	1,41230607	1,53998338	1,65587664	1,87643408	1,96549518	2,04779527
6,5	1,81948246	0,0903802	2,28306515	1,46745979	1,59077324	1,70468562	1,92566379	2,0161923	2,10041675
7	1,86819559	0,08827037	2,14337272	1,521791	1,64148036	1,75370936	1,97517143	2,06710781	2,15318932
7,5	1,91608275	0,08632379	2,01923481	1,57404601	1,69088885	1,8017894	2,02381511	2,1170503	2,20484351
8	1,96193422	0,08459003	1,91820944	1,62289821	1,73770156	1,84768139	2,07037956	2,16476266	2,25404914
8,5	2,0048452	0,08310468	1,84688948	1,66733777	1,78092969	1,89044264	2,11396712	2,20932235	2,29982963
9	2,04449178	0,0818732	1,80967805	1,7069972	1,82020061	1,92972344	2,15426439	2,25040598	2,34183157
9,5	2,08084164	0,08088994	1,80822368	1,74183823	1,85545719	1,96547365	2,19125576	2,28800687	2,38004875
10	2,11398881	0,08015408	1,84063335	1,7719636	1,88677421	1,99776717	2,22507025	2,32229475	2,41469672
10,5	2,14402799	0,07966389	1,90175387	1,79753335	1,91425959	2,02669028	2,25584039	2,353459	2,44603915
11	2,17079908	0,07939852	1,98262533	1,81867541	1,93789576	2,05213085	2,28340984	2,38138617	2,47403721
11,5	2,19405784	0,07933119	2,07186623	1,83556595	1,95764894	2,07391859	2,30752733	2,4058677	2,49856325
12	2,21367878	0,07943541	2,156923	1,84856846	1,97362725	2,09200568	2,32806629	2,42682889	2,51963346
12,5	2,22979316	0,07968401	2,22608239	1,85830884	1,98618581	2,10659289	2,34516438	2,4444622	2,53752908
13	2,24279612	0,08004762	2,27097047	1,86559704	1,9959004	2,11812933	2,35922292	2,45921091	2,5527589
13,5	2,25287664	0,08049696	2,28675709	1,87098658	2,00312883	2,12686188	2,37041601	2,47126042	2,56553541
14	2,26006442	0,08100323	2,27007845	1,87485503	2,00807203	2,13288426	2,37874669	2,4806119	2,57587296
14,5	2,26434213	0,08154664	2,22082233	1,87735577	2,01080875	2,13622049	2,38417151	2,4872086	2,58370967
15	2,26572708	0,08212306	2,1437421	1,878369	2,0113125	2,13688271	2,38669043	2,49102343	2,58898577
15,5	2,2642984	0,08273659	2,04548703	1,87768461	2,00954634	2,13491936	2,38637326	2,49209625	2,59169921
16	2,26011313	0,08339291	1,93279685	1,87505623	2,0054433	2,13035414	2,38326692	2,49044095	2,5918172
16,5	2,25330035	0,08409325	1,81144251	1,87036302	1,99902976	2,1232891	2,37748935	2,48614375	2,58938179
17	2,24419268	0,08483235	1,68534916	1,86376237	1,99056259	2,11403516	2,36936855	2,47951361	2,5846744
17,5	2,23333916	0,08560092	1,55674273	1,85567697	1,98052866	2,1031191	2,35946057	2,47110469	2,57824318
18	2,22143528	0,08638951	1,42718112	1,84667664	1,96955365	2,09120712	2,34847882	2,46164063	2,57081901

Table S2. Reference centiles for V_{max} in the S2LJ in healthy males in Germany, in the age of 6-18 years

age	mu	sigma	lambda	C3	C10	C25	C75	C90	C97
6	1,74928138	0,09725158	3,0048854	1,34167361	1,49674853	1,62692198	1,85653459	1,94440251	2,02368882
6,5	1,80050852	0,09556595	2,83984616	1,40034584	1,54928253	1,67766042	1,90960919	1,99982982	2,08180827
7	1,85195124	0,09395803	2,6795132	1,45784935	1,60170717	1,72856933	1,96287999	2,05543929	2,14011698
7,5	1,90235274	0,09244928	2,52800907	1,51311668	1,65281677	1,7784221	2,01503431	2,10984882	2,19715249
8	1,95044529	0,09105117	2,38942292	1,56508095	1,70141411	1,82598889	2,06474261	2,16165245	2,25141868
8,5	1,99523663	0,08976794	2,2664958	1,61294919	1,74657624	1,8703086	2,1109667	2,20975339	2,30174684
9	2,03648757	0,08860593	2,15896901	1,65661195	1,78807545	1,91113291	2,15347925	2,25392877	2,34791021
9,5	2,07448526	0,08757627	2,0658026	1,69635959	1,82613979	1,94870389	2,19261861	2,29456022	2,39032713
10	2,10992905	0,08669574	1,98669182	1,73274264	1,86133088	1,98364127	2,22916333	2,33249214	2,42989891
10,5	2,14388425	0,08598104	1,92167445	1,76659084	1,89453027	2,01690982	2,26427652	2,36896971	2,46794342
11	2,17761629	0,08543489	1,87022234	1,79897993	1,92684986	2,04968806	2,29931383	2,40542883	2,50597162
11,5	2,21239111	0,08504932	1,83147242	1,83103878	1,95943989	2,08317428	2,33561537	2,44327851	2,54545937
12	2,24936048	0,08480807	1,8043467	1,86383684	1,99337659	2,11847384	2,37438741	2,48377804	2,58771938
12,5	2,28917635	0,08468401	1,78770123	1,89807291	2,02932604	2,15623919	2,41629104	2,52760228	2,63344333
13	2,33144175	0,08463567	1,7805248	1,93363531	2,06706799	2,19615848	2,46085598	2,57422186	2,68204925
13,5	2,37495986	0,08461764	1,78191286	1,96976765	2,10568884	2,23717575	2,50675726	2,62220404	2,73200523
14	2,41821113	0,08458746	1,79087479	2,0054459	2,1439893	2,27793124	2,5523296	2,66976094	2,78141079
14,5	2,45974112	0,08451355	1,8063033	2,03966506	2,18080376	2,3171137	2,5959898	2,71520275	2,82847969
15	2,49839297	0,08438051	1,82722613	2,07158964	2,21518038	2,35366712	2,63649207	2,75721147	2,87182997
15,5	2,53334145	0,08418526	1,85298031	2,1005879	2,24641665	2,38682618	2,6729594	2,79486904	2,91050811
16	2,5640626	0,08393028	1,88253066	2,12626489	2,27406403	2,41610061	2,70484462	2,82761482	2,94394628
16,5	2,59062282	0,08362799	1,91459457	2,14866922	2,29816444	2,44153837	2,73224248	2,85557634	2,97230963
17	2,6136715	0,08329686	1,94823932	2,1682796	2,31924542	2,46372256	2,75587288	2,87954053	2,99645316
17,5	2,63420479	0,08295317	1,98312896	2,18584454	2,33813675	2,48356326	2,77681615	2,90066295	3,01760518
18	2,65337218	0,08260807	2,01889261	2,20226776	2,35582254	2,50212434	2,7963056	2,92025177	3,03714624

Table S3. Reference centiles for H_{max} in the S2LJ in healthy females in Germany, in the age of 6-18 years

age	mu	sigma	lambda	C3	C10	C25	C75	C90	C97
6	0,22957323	0,16894348	0,72900286	0,15996416	0,18143459	0,20399303	0,255951	0,28062737	0,3054875
6,5	0,24104301	0,1643578	0,63945069	0,17083987	0,19229565	0,21503189	0,26810827	0,29364046	0,3195609
7	0,25281948	0,16003542	0,55423477	0,18191378	0,20341322	0,22636163	0,2805731	0,30695592	0,33393226
7,5	0,26461063	0,15605752	0,47850297	0,19289021	0,21449872	0,23769649	0,29303335	0,32022835	0,34820747
8	0,27606666	0,15249547	0,41879546	0,2034103	0,22520399	0,24869321	0,30511502	0,33304184	0,36190705
8,5	0,28688368	0,14939581	0,38193288	0,21314576	0,2352167	0,25904923	0,31649458	0,34503391	0,37460573
9	0,29692142	0,14675811	0,37381334	0,22191035	0,24437297	0,26861711	0,32702331	0,35603027	0,38608329
9,5	0,30615038	0,14456919	0,39787627	0,22962264	0,25261374	0,27735526	0,33667495	0,36599866	0,396292
10	0,3145911	0,14283077	0,45376976	0,23625909	0,25993184	0,28527029	0,34548554	0,37499344	0,40531091
10,5	0,32227107	0,14155007	0,53761019	0,24182329	0,26633802	0,29237911	0,35350336	0,38309655	0,41327447
11	0,32916183	0,14071107	0,64095003	0,24633135	0,27182726	0,29865961	0,3607147	0,39033301	0,42027556
11,5	0,33519969	0,14028944	0,75236823	0,24981973	0,2763889	0,3040656	0,36706746	0,39669164	0,42637514
12	0,34033107	0,14026181	0,85942934	0,25236936	0,28003646	0,30856451	0,37252049	0,40217247	0,4316416
12,5	0,34455857	0,14060304	0,95092167	0,25412463	0,28283777	0,31217702	0,37709038	0,4068296	0,43618588
13	0,34797525	0,14127931	1,01906047	0,25529226	0,28493232	0,31500617	0,3808848	0,41079999	0,44018427
13,5	0,35067367	0,14225137	1,05955025	0,25605765	0,28645006	0,31715311	0,38400418	0,4142002	0,44377369
14	0,35271441	0,14347434	1,06971613	0,2565733	0,28749248	0,31869075	0,3865104	0,41710105	0,44703818
14,5	0,35411012	0,14490507	1,05011136	0,25690693	0,28810196	0,31964407	0,3884086	0,41950361	0,44997645
15	0,35482714	0,14650635	1,00630526	0,25700234	0,28824497	0,31998641	0,38964626	0,42133014	0,45247994
15,5	0,35481582	0,1482389	0,94647889	0,25674046	0,28785406	0,31967206	0,39014716	0,4224647	0,45438222
16	0,35402187	0,15006197	0,87912493	0,2559872	0,28685266	0,31865	0,38982678	0,42277599	0,45549108
16,5	0,35243266	0,15193529	0,81118586	0,25465884	0,28520561	0,31690855	0,38864688	0,42218365	0,45566935
17	0,3501269	0,1538331	0,74610311	0,25277996	0,28296739	0,31452038	0,38667828	0,42073994	0,45494122
17,5	0,34727909	0,15575095	0,68386567	0,2504816	0,28028316	0,31164506	0,38410907	0,4186441	0,45351589
18	0,34411755	0,15769101	0,62358418	0,24794178	0,27734388	0,3084899	0,38119139	0,41617235	0,45169567

Table S4. Reference centiles for H_{max} in the S2LJ in healthy males in Germany, in the age of 6-18 years

age	mu	sigma	lambda	C3	C10	C25	C75	C90	C97
6	0,22683905	0,1900449	0,96950778	0,14629451	0,1718811	0,19801407	0,25577645	0,28221	0,30828421
6,5	0,23883522	0,18600216	0,88471265	0,15717948	0,18280605	0,20929225	0,26880652	0,29643468	0,32390315
7	0,25114457	0,1821556	0,80297429	0,16827029	0,19400206	0,22087182	0,28215512	0,3109876	0,3398715
7,5	0,26348029	0,17854081	0,72809852	0,17930663	0,20520718	0,2324844	0,29550199	0,32550386	0,35576713
8	0,27553202	0,1751677	0,66404507	0,19001185	0,21614094	0,24384086	0,30849866	0,339583	0,37112083
8,5	0,28702947	0,17202825	0,61290403	0,20016316	0,22656714	0,25469134	0,32084394	0,35288317	0,38553663
9	0,2978638	0,16912286	0,57274287	0,20969584	0,23639761	0,26493376	0,33242805	0,36529635	0,3989087
9,5	0,30805777	0,16646583	0,54049173	0,218643	0,24565051	0,27458257	0,34329393	0,37689308	0,41134237
10	0,31772732	0,16408332	0,51424652	0,22708154	0,25440711	0,28373135	0,35358863	0,38785592	0,42306131
10,5	0,32706176	0,16200119	0,4937229	0,23512055	0,26279852	0,29253714	0,36353712	0,39844475	0,4343615
11	0,33631441	0,16022568	0,48003737	0,24290897	0,27100746	0,30121683	0,37342722	0,40897624	0,44558483
11,5	0,34577711	0,15874319	0,47450393	0,25063367	0,27925596	0,31002681	0,38358281	0,41979968	0,45710142
12	0,35574782	0,15752331	0,47828589	0,25849783	0,2877799	0,31923405	0,3943289	0,43126069	0,46927277
12,5	0,36642025	0,15651841	0,49210563	0,26664339	0,29674114	0,32901747	0,40586963	0,44356889	0,48231009
13	0,37772288	0,15566117	0,51594606	0,27503945	0,30610016	0,33932452	0,41811001	0,45660453	0,49606652
13,5	0,3893526	0,15488033	0,54935078	0,28349578	0,3156359	0,34989753	0,43069718	0,46996669	0,5100923
14	0,4009124	0,15411177	0,59171863	0,2917553	0,32505187	0,36039443	0,44317573	0,48314531	0,52382358
14,5	0,41202762	0,15331002	0,64174871	0,29958223	0,33407002	0,37049153	0,45512147	0,49567429	0,53675775
15	0,42241052	0,152456	0,69742032	0,30680559	0,34247949	0,37993918	0,46621592	0,5072155	0,54854659
15,5	0,43184108	0,15154769	0,75684153	0,31329647	0,35011772	0,38854427	0,47622108	0,51752381	0,5589476
16	0,4401655	0,1505893	0,81814696	0,31897444	0,35687419	0,39617155	0,48497493	0,52643943	0,56781262
16,5	0,44738009	0,14959695	0,88003851	0,32385204	0,36274971	0,40281592	0,49248397	0,53398585	0,57518885
17	0,4536403	0,14859183	0,94213017	0,32803613	0,36786307	0,40861155	0,4989296	0,54037188	0,58131524
17,5	0,45920762	0,14759016	1,00450395	0,33170321	0,37241672	0,41378822	0,50460676	0,54592425	0,58655065
18	0,46440083	0,14660297	1,06699527	0,33507795	0,37666395	0,41862955	0,50987134	0,55103397	0,59132156

Table S5. Reference centiles for P_{max}/mass in the S2LJ in healthy females in Germany, in the age of 6-18 years

age	mu	sigma	lambda	C3	C10	C25	C75	C90	C97
6	28,9798604	0,13893234	0,23230876	22,1347371	24,1670458	26,3770748	31,7754385	34,4965494	37,3441514
6,5	29,8894541	0,1385364	0,16118626	22,9067674	24,9680134	27,2208976	32,7740683	35,5999898	38,5762028
7	30,8082463	0,13816319	0,09637573	23,6823287	25,775104	28,0727023	33,7824433	36,713373	39,8184862
7,5	31,7168267	0,13783385	0,04378696	24,4404225	26,5687318	28,9135821	34,7788629	37,8108724	41,0387163
8	32,5958513	0,13758073	0,00837669	25,1599297	27,329089	29,7244614	35,742074	38,8675064	42,2057722
8,5	33,4319816	0,13743245	-0,0066762	25,8255045	28,0419795	30,4919137	36,6576094	39,8662415	43,2981139
9	34,2214393	0,13739454	-0,0011502	26,4323583	28,7030991	31,2120069	37,521404	40,8021645	44,3092051
9,5	34,9646826	0,13746281	0,02301635	26,9811036	29,3129336	31,8851584	38,3341478	41,6764222	45,2409665
10	35,6635847	0,13763703	0,06230928	27,4746523	29,8737638	32,5132392	39,0984575	42,4932644	46,100506
10,5	36,3201242	0,13791242	0,1124581	27,9171607	30,3886238	33,0984752	39,8171016	43,2574946	46,8963244
11	36,9319469	0,13826037	0,16939546	28,3110241	30,858022	33,6397366	40,4874522	43,9674406	47,6292273
11,5	37,4920269	0,13864427	0,22946684	28,655825	31,2790661	34,1318962	41,1014656	44,6152071	48,2927834
12	37,991914	0,13902552	0,28905066	28,9510109	31,6481823	34,5687232	41,6494662	45,1911123	48,8785683
12,5	38,4251525	0,139362	0,34469404	29,1984831	31,9639593	34,9460025	42,123833	45,6875739	49,3801965
13	38,7885771	0,13960823	0,39395162	29,4024734	32,2276726	35,2625449	42,5202791	46,1000816	49,7937022
13,5	39,0767694	0,13971757	0,43568001	29,5644963	32,4382402	35,5149919	42,8317807	46,4205115	50,1101607
14	39,2845885	0,13964928	0,46897379	29,6857281	32,5944479	35,7000605	43,0517403	46,6412386	50,3211483
14,5	39,4092616	0,13938915	0,49383874	29,7661455	32,6951914	35,8157572	43,1767434	46,7583091	50,4221426
15	39,4517987	0,13896676	0,51170536	29,8035501	32,7394084	35,8620934	43,208507	46,7737329	50,4150221
15,5	39,41715	0,13843986	0,52400551	29,7963903	32,7277465	35,8418554	43,1538683	46,6957506	50,3090478
16	39,313384	0,13787495	0,53146665	29,7454457	32,6632925	35,7605074	43,0234462	46,5372138	50,1191458
16,5	39,1515976	0,13732251	0,53477121	29,6547525	32,5523672	35,6266731	42,8307142	46,3136442	49,8627177
17	38,9449005	0,13680253	0,53495519	29,531438	32,4041974	35,4515424	42,590417	46,0409969	49,5566228
17,5	38,706813	0,13631174	0,53326263	29,3842942	32,2293688	35,2472621	42,3170552	45,7342924	49,2160221
18	38,4510668	0,1358411	0,53072964	29,2229963	32,0390855	35,0263737	42,0252299	45,4086221	48,8561897

Table S6. Reference centiles for P_{max}/mass in the S2LJ in healthy males in Germany, in the age of 6-18 years

age	mu	sigma	lambda	C3	C10	C25	C75	C90	C97
6	28,1399876	0,14691657	1,02501417	20,3377384	22,8348165	25,3665192	30,9066237	33,4201038	35,8877686
6,5	29,1629385	0,14647588	0,97830187	21,1586392	23,7070593	26,304069	32,0279116	34,6411836	37,2158004
7	30,1968993	0,14608789	0,93257494	21,9872222	24,5880014	27,2513015	33,1620272	35,8773159	38,5616946
7,5	31,2173381	0,1457634	0,88825535	22,8041955	25,4567973	28,1856809	34,2823008	37,0997728	39,8946081
8	32,2000518	0,14549813	0,84561392	23,5912845	26,2933939	29,0852426	35,362143	38,2796686	41,1832906
8,5	33,1284309	0,1452808	0,8050532	24,335878	27,0840799	29,9349939	36,3831001	39,3966752	42,4053944
9	34,001981	0,14510315	0,76726389	25,0366418	27,8280305	30,7344052	37,3443926	40,4494681	43,5587935
9,5	34,8307075	0,14496265	0,73322274	25,6993344	28,5326459	31,4922849	38,256823	41,4492073	44,654645
10	35,6306737	0,14486744	0,70390006	26,3336186	29,2098731	32,2226886	39,1380866	42,4147116	45,7125104
10,5	36,4215058	0,14483374	0,67996049	26,9517096	29,8744142	32,9427499	40,0100555	43,369638	46,7575259
11	37,2227089	0,14486989	0,66173068	27,5666242	30,5413914	33,6697161	40,8943565	44,3373825	47,8146417
11,5	38,0543579	0,14497376	0,64933923	28,1926473	31,2269429	34,4216184	41,8130238	45,3416529	48,9090782
12	38,9359322	0,14513336	0,64282502	28,8442859	31,9471855	35,2161988	42,78719	46,4049854	50,0646341
12,5	39,8785961	0,14532342	0,64229282	29,5305813	32,7119646	36,0640055	43,8284496	47,5391857	51,2931398
13	40,8752099	0,14549959	0,64802911	30,2480352	33,516938	36,9595358	44,9276367	48,7328977	52,5807576
13,5	41,904994	0,14561077	0,66034666	30,9836379	34,347049	37,8852337	46,0602567	49,9578257	53,8950727
14	42,9419706	0,14561112	0,67939768	31,7207191	35,1830829	38,8188776	47,1961373	51,1796294	55,197347
14,5	43,9616613	0,14547272	0,70491288	32,4434528	36,0067099	39,7393238	48,3072793	52,3669972	56,4529586
15	44,9447482	0,14519279	0,73611679	33,1391013	36,803086	40,6295639	49,3722187	53,4967258	57,6372503
15,5	45,8770014	0,14478404	0,77187271	33,7983237	37,5610705	41,476825	50,3757166	54,5531193	58,7345619
16	46,7487162	0,14426401	0,81083178	34,4152584	38,2731536	42,2722979	51,3078115	55,526594	59,7362895
16,5	47,5587697	0,14365786	0,85201866	34,9891637	38,9380868	43,014567	52,1682274	56,4181605	60,6452093
17	48,315446	0,14299337	0,89509135	35,5247565	39,5615411	43,7104317	52,9670319	57,2397929	61,4754452
17,5	49,0343425	0,14229485	0,93989344	36,0318723	40,1550932	44,3733143	53,7221918	58,0118028	62,2497964
18	49,7354435	0,14158446	0,98574885	36,5245324	40,7343735	45,0207521	54,4565222	58,7599153	62,9971527

Table S7. Reference centiles for F_{max}/BW in the S2LJ in healthy females in Germany, in the age of 6-18 years

age	mu	sigma	lambda	C3	C10	C25	C75	C90	C97
6	22,464226	0,16815551	-1,218537	17,1930718	18,5557418	20,2135135	25,3584392	28,8340405	33,4872384
6,5	22,3123391	0,16489119	-1,2786881	17,1847405	18,5096034	20,1217468	25,1345399	28,5350231	33,1133754
7	22,1649082	0,16170103	-1,3340581	17,1728051	18,4624998	20,0317563	24,9163988	28,2396118	32,7331577
7,5	22,0272065	0,15858473	-1,381356	17,160366	18,418287	19,9481456	24,7094476	27,9521105	32,3463918
8	21,9046007	0,15555754	-1,4187015	17,1511806	18,3811656	19,8756289	24,5197951	27,6792818	31,9599501
8,5	21,8020834	0,15264421	-1,4460085	17,1494016	18,3553834	19,8186732	24,3535792	27,4294602	31,5866998
9	21,7235453	0,14986183	-1,4653559	17,1593566	18,3449076	19,7810204	24,2158158	27,2106987	31,2422783
9,5	21,6711848	0,14721786	-1,479751	17,1844499	18,3525662	19,7650272	24,1094781	27,0286547	30,9396005
10	21,6462336	0,14471292	-1,4920831	17,2272172	18,3803425	19,7721937	24,0362227	26,8868458	30,6874267
10,5	21,6492431	0,14234764	-1,5045935	17,2892308	18,4293959	19,8033094	23,996802	26,7871203	30,4907318
11	21,677812	0,14013505	-1,5182038	17,3687915	18,4977803	19,8561881	23,9887327	26,7271936	30,3480047
11,5	21,7260646	0,13809833	-1,5330806	17,4608028	18,5801699	19,9252529	24,0058137	26,7005321	30,2522957
12	21,7865626	0,13625658	-1,5490994	17,5588102	18,6698485	20,0034779	24,0400692	26,6984697	30,1938609
12,5	21,8523332	0,13460914	-1,5658521	17,6570226	18,7607519	20,0844391	24,0836997	26,7119977	30,1616298
13	21,917509	0,13312627	-1,5823921	17,7510039	18,8481743	20,1630113	24,1295664	26,7319382	30,1427383
13,5	21,9756436	0,13176987	-1,5975324	17,8359625	18,9270158	20,23361	24,1697458	26,7480089	30,1226738
14	22,0209239	0,13050326	-1,6102881	17,9076619	18,9927321	20,2912246	24,1970908	26,7509872	30,0885808
14,5	22,0498125	0,12928821	-1,6199376	17,9638499	19,0428222	20,3329741	24,2069761	26,734569	30,0312529
15	22,0620449	0,12808376	-1,6260275	18,0051145	19,0777245	20,359048	24,1983591	26,6964936	29,9463291
15,5	22,0598389	0,12685595	-1,6285683	18,0340797	19,0999917	20,3718791	24,1731066	26,6381205	29,8343977
16	22,0471871	0,12559026	-1,6283613	18,0546292	19,1134993	20,375364	24,1354733	26,564269	29,7015474
16,5	22,0289605	0,12428693	-1,626419	18,0710849	19,1226476	20,374055	24,090954	26,4815394	29,5565417
17	22,0090826	0,12295652	-1,6234771	18,0866551	19,1307725	20,3714953	24,0441429	26,3956249	29,4070862
17,5	21,9892186	0,12161525	-1,6199084	18,1024403	19,1390782	20,3690543	23,9972122	26,3095019	29,2575772
18	21,9694106	0,12028022	-1,6160076	18,1181524	19,1473239	20,3665841	23,950557	26,2241484	29,1100348

Table S8. Reference centiles for F_{max}/BW in the S2LJ in healthy males in Germany, in the age of 6-18 years

age	mu	sigma	lambda	C3	C10	C25	C75	C90	C97
6	21,3307094	0,1472927	-1,0319453	16,7195972	17,9551198	19,4174015	23,6704744	26,306429	29,5552383
6,5	21,2886235	0,14430507	-1,0419639	16,7642537	17,9796762	19,4151617	23,5725114	26,136386	29,2834691
7	21,2447013	0,14141058	-1,0539747	16,8060711	18,0013103	19,4102236	23,4747117	25,9701012	29,0219897
7,5	21,1988679	0,13861383	-1,0693321	16,8453005	18,0200966	19,4025365	23,3771863	25,8081722	28,7723784
8	21,1526777	0,13591908	-1,0889727	16,8832637	18,0373748	19,3935044	23,2817629	25,6528304	28,5374656
8,5	21,1091876	0,13333678	-1,1132222	16,9220774	18,0555152	19,3857992	23,1919365	25,5079951	28,3215509
9	21,0721071	0,13089235	-1,1412708	16,9636323	18,0769613	19,382461	23,1120366	25,3784001	28,1293953
9,5	21,0458459	0,12861542	-1,1718025	17,0102047	18,1046479	19,3871256	23,0471762	25,2696041	27,9665732
10	21,0353631	0,12653358	-1,2035873	17,0647273	18,1421214	19,4040014	23,0030417	25,187847	27,8396051
10,5	21,0452223	0,12466876	-1,2357466	17,1301773	18,192827	19,4370479	22,9848243	25,138878	27,75471
11	21,0779731	0,12302452	-1,2678733	17,208564	18,2589447	19,488622	22,9952525	25,1255584	27,7148242
11,5	21,1352604	0,12158904	-1,2997004	17,3015958	18,3421953	19,5604309	23,0358335	25,1491672	27,7208561
12	21,2177691	0,12034435	-1,330968	17,4103428	18,4435777	19,6533579	23,1069407	25,2096461	27,7721113
12,5	21,3240851	0,11927304	-1,3615155	17,5341485	18,5622542	19,7663296	23,2067001	25,3045532	27,8653901
13	21,4493648	0,11835822	-1,391405	17,6695545	18,6944004	19,8950828	23,3295545	25,4275324	27,9933738
13,5	21,5868367	0,11758002	-1,4206275	17,8115197	18,834524	20,0335788	23,4678131	25,5698312	28,1459765
14	21,7293781	0,11691666	-1,4491626	17,9547173	18,9768333	20,1754598	23,6133811	25,7222169	28,3125061
14,5	21,8709797	0,11634773	-1,4770974	18,0947079	19,1164793	20,3153815	23,7594135	25,8768759	28,4839245
15	22,007243	0,11585804	-1,5046558	18,228276	19,2499294	20,4494357	23,900918	26,0281605	28,6538086
15,5	22,1345911	0,11543898	-1,5321552	18,3527346	19,3742471	20,5744001	24,0339181	26,1716996	28,8174013
16	22,2501483	0,11508421	-1,5601619	18,4659866	19,4871013	20,6876896	24,1552584	26,3041394	28,9713105
16,5	22,352269	0,11478763	-1,5890262	18,566883	19,5871981	20,7878487	24,2631008	26,4234946	29,1135062
17	22,4414072	0,11454361	-1,6187054	18,6558958	19,6750145	20,8753511	24,357857	26,5301127	29,244256
17,5	22,5205363	0,11434468	-1,6489107	18,7355779	19,7532422	20,953028	24,442611	26,6271217	29,3666326
18	22,5942454	0,11417651	-1,6793284	18,810012	19,8261359	21,0253034	24,5220631	26,7192231	29,4851542

Table S9. Reference centiles for the Nerve-Muscle Index in the S2LJ in healthy females in Germany, in the age of 6-18 years

age	mu	sigma	nu	C3	C10	C25	C75	C90	C97
6	80,7972173	0,19899589	1,91042352	41,9051152	56,9941726	69,2732187	90,9868482	99,4334261	107,147619
6,5	83,0432852	0,19229462	1,95232903	44,3754895	59,3755382	71,590379	93,1569624	101,530214	109,169004
7	85,3138101	0,18590843	1,99354494	46,8893138	61,7872835	73,9327883	95,3535964	103,656953	111,224702
7,5	87,5632657	0,17988959	2,03386558	49,3993802	64,1849366	76,255951	97,5297784	105,765191	113,264732
8	89,7404742	0,17429254	2,07354861	51,8448896	66,5152838	78,508764	99,6331	107,800865	115,233108
8,5	91,7994708	0,16916664	2,11297911	54,1653356	68,7272956	80,6439224	101,617531	109,717205	117,08194
9	93,7065927	0,16453436	2,15261488	56,3134942	70,7820213	82,6261328	103,449771	111,48061	118,776959
9,5	95,4372804	0,16039826	2,19298067	58,2531129	72,6501935	84,4293424	105,10565	113,066709	120,293269
10	96,9716451	0,1567528	2,2345565	59,9545399	74,30795	86,0323781	106,565664	114,455898	121,610906
10,5	98,294822	0,15358893	2,27754791	61,3958725	75,7373075	87,4192262	107,815563	115,634228	122,716063
11	99,4053677	0,15089066	2,32156573	62,5724361	76,9343691	88,587083	108,855349	116,603054	123,611443
11,5	100,317476	0,14864161	2,36597435	63,4926235	77,9089644	89,5477218	109,701769	117,381489	124,318494
12	101,054872	0,14682208	2,41013528	64,1729812	78,6798387	90,3221905	110,381284	117,998486	124,868598
12,5	101,644411	0,14539923	2,45357697	64,6362883	79,2710119	90,935761	110,92245	118,48419	125,293466
13	102,113292	0,14431788	2,49620413	64,9116132	79,7107807	91,4160306	111,352315	118,86564	125,620201
13,5	102,489892	0,14351801	2,5383688	65,0279273	80,0288888	91,7921747	111,698457	119,169704	125,874993
14	102,797759	0,14293914	2,58051803	65,011836	80,2518998	92,0893378	111,982934	119,417175	126,077455
14,5	103,047728	0,14251951	2,62293298	64,8855643	80,3978722	92,321719	112,213828	119,613983	126,231635
15	103,232872	0,14219436	2,66567035	64,665682	80,4729325	92,4881702	112,379744	119,745374	126,319928
15,5	103,334471	0,1418981	2,70878353	64,36456	80,4749331	92,577214	112,456404	119,782904	126,310356
16	103,327185	0,14157702	2,75261878	63,9850033	80,393958	92,570405	112,412976	119,691497	126,164154
16,5	103,191369	0,14119631	2,79766207	63,5255341	80,2207119	92,4528025	112,225978	119,444653	125,852161
17	102,929247	0,14074824	2,84415876	62,9897218	79,9582987	92,2269423	111,897488	119,044387	125,376371
17,5	102,56937	0,14024925	2,8918642	62,391807	79,6267535	91,9171825	111,459827	118,52617	124,775132
18	102,153451	0,1397256	2,94025363	61,7518949	79,2549633	91,5590027	110,960226	117,941784	124,104365

Table S10. Reference centiles for the Nerve-Muscle Index in the S2LJ in healthy males in Germany, in the age of 6-18 years

age	mu	sigma	nu	C3	C10	C25	C75	C90	C97
6	82,6478741	0,18164913	1,76140614	49,019703	61,2812361	72,0681643	92,2827464	100,431097	107,981813
6,5	84,9398796	0,17411378	1,74744646	52,2638269	64,0456184	74,5511698	94,4540072	102,530881	110,035809
7	87,2627317	0,16694329	1,73779182	55,4380017	66,8077635	77,0572508	96,6538107	104,6523	112,1019
7,5	89,5819386	0,16015774	1,73533598	58,5092595	69,5324888	79,5521846	98,8455009	106,755289	114,135576
8	91,8587699	0,15377936	1,74220417	61,4457921	72,1824917	81,9977699	100,989183	108,798183	116,09281
8,5	94,0558686	0,14783145	1,75918606	64,2212382	74,7231754	84,3570217	103,047934	110,744671	117,937919
9	96,1491379	0,14234231	1,78504453	66,8234175	77,1322624	86,6048459	105,000992	112,578259	119,659379
9,5	98,1221378	0,13734051	1,81791234	69,2425724	79,3926416	88,7226912	106,836325	114,292236	121,256561
10	99,9639751	0,13285371	1,85621024	71,4666346	81,4894508	90,6964875	108,547692	115,885448	122,733884
10,5	101,673768	0,12890255	1,89847777	73,4886918	83,415738	92,5214792	110,139037	117,366784	124,105465
11	103,268011	0,12548416	1,94289323	75,3203804	85,1827033	94,2106612	111,631366	118,761954	125,402043
11,5	104,768333	0,12257593	1,98746105	76,9825333	86,8094188	95,7835746	113,049453	120,099214	126,655708
12	106,197171	0,12014547	2,03024811	78,4992308	88,3176133	97,2616073	114,417728	121,405364	127,895947
12,5	107,57244	0,11815373	2,06969007	79,8923108	89,7264149	98,6627733	115,754747	122,699958	129,143573
13	108,900072	0,11655316	2,10481938	81,1757567	91,0461426	99,9948986	117,065094	123,986677	130,401584
13,5	110,174766	0,11529533	2,13484471	82,3561717	92,2783763	101,255833	118,340956	125,255788	131,658604
14	111,383915	0,11433254	2,15901326	83,4357964	93,4191203	102,436922	119,566544	126,489068	132,894326
14,5	112,510281	0,11361838	2,17671096	84,4138726	94,4607574	103,525374	120,721013	127,662884	134,082713
15	113,537109	0,11311057	2,18740993	85,2897095	95,3958566	104,508717	121,784167	128,75441	135,198696
15,5	114,455533	0,11277455	2,19046823	86,0678077	96,2229951	105,381367	122,744719	129,75067	136,22798
16	115,26356	0,11258014	2,18567398	86,7554491	96,945494	106,143733	123,598823	130,646462	137,164265
16,5	115,972396	0,11250155	2,17360307	87,3656148	97,5760248	106,807864	124,356774	131,451226	138,016136
17	116,605829	0,11251704	2,1557245	87,9159761	98,1358583	107,396814	125,042303	132,188197	138,806055
17,5	117,191454	0,11260423	2,13402835	88,4252705	98,6488789	107,937078	125,682959	132,88422	139,55974
18	117,752601	0,11273538	2,11042902	88,91116	99,1365676	108,451753	126,301287	133,560536	140,296768

Table S11. Reference centiles for the side-difference of the impulse in the S2LJ in healthy females in Germany, in the age of 6-18 years

age	mu	sigma	nu	C3	C10	C25	C75	C90	C97
6	6,93029726	0,58652028	0,22881116	1,94485279	3,04100221	4,59115268	10,0965792	13,8559516	18,5258816
6,5	6,74586351	0,58197354	0,21816176	1,93257687	2,99215864	4,48804734	9,80754043	13,4526394	17,9953503
7	6,56765472	0,57747685	0,20737808	1,91968024	2,94404617	4,38785929	9,52888282	13,0641333	17,4845276
7,5	6,39634343	0,57308703	0,1960351	1,90687295	2,89724147	4,29108048	9,2620967	12,6936509	16,9997606
8	6,23340573	0,56888637	0,18365946	1,89499325	2,8525906	4,19868295	9,00993169	12,3462637	16,550108
8,5	6,08129524	0,564966	0,17001449	1,88477325	2,81113882	4,11221266	8,77637474	12,0282366	16,1453501
9	5,94279689	0,56145656	0,15545267	1,87615684	2,77348986	4,0331856	8,56571562	11,745382	15,7929025
9,5	5,82096881	0,55849473	0,14068044	1,86890882	2,74022131	3,96325782	8,38246262	11,5033102	15,4989209
10	5,71914211	0,55618813	0,12648387	1,86314571	2,71225604	3,90440178	8,23134409	11,3077639	15,269486
10,5	5,6396191	0,55459596	0,11355025	1,85912596	2,69038936	3,85808329	8,11545739	11,162262	15,1078269
11	5,58110771	0,55368195	0,10227275	1,85666582	2,67427194	3,8236289	8,0324537	11,0628542	15,0074248
11,5	5,5396751	0,55334815	0,09285603	1,85504048	2,66255007	3,79870677	7,97602634	11,0000892	14,954368
12	5,51036667	0,55346499	0,08541634	1,85325173	2,65343667	3,7803245	7,93831555	10,9622682	14,9315389
12,5	5,48967887	0,55391871	0,08002121	1,85065817	2,64572634	3,76640814	7,91366652	10,9408342	14,9261815
13	5,47724072	0,55466677	0,07669469	1,84737224	2,63944033	3,75683133	7,90132688	10,9344645	14,9360064
13,5	5,47279166	0,55572517	0,07544841	1,84328253	2,63441451	3,75137735	7,90101142	10,9428433	14,9605775
14	5,47515133	0,55712217	0,07631806	1,83784767	2,6299484	3,74914613	7,91115558	10,9638951	14,9970459
14,5	5,48301234	0,55884171	0,07923952	1,83072134	2,62548198	3,74928033	7,92983037	10,9949309	15,0417482
15	5,49571721	0,56078364	0,08391479	1,82232575	2,62121657	3,75164138	7,95573196	11,0340359	15,0923363
15,5	5,51353599	0,56278005	0,08986524	1,81384118	2,61816486	3,75694862	7,98864519	11,0807365	15,1486451
16	5,53828714	0,56466217	0,09657966	1,80700434	2,61810923	3,76699215	8,0306152	11,137773	15,2150721
16,5	5,57147367	0,56631253	0,10360845	1,80319629	2,6224641	3,78320187	8,08346629	11,207795	15,2960722
17	5,61265054	0,56772466	0,11067313	1,80256826	2,6312154	3,80535435	8,14667632	11,2905575	15,3923333
17,5	5,65965241	0,56899239	0,11768013	1,80413988	2,64305356	3,83174964	8,21764327	11,3831922	15,5010039
18	5,71056965	0,57021182	0,12461654	1,80692121	2,65672601	3,86083405	8,29410607	11,4831668	15,6193895

Table S12. Reference centiles for the side-difference of the impulse in the S2LJ in healthy males in Germany, in the age of 6-18 years

age	mu	sigma	nu	C3	C10	C25	C75	C90	C97
6	7,00594286	0,55551721	0,1103957	2,30968892	3,3408856	4,79081015	10,0896681	13,8917043	18,8250257
6,5	6,88499729	0,55816264	0,09400486	2,28089136	3,28633647	4,70508627	9,94321487	13,747047	18,7295973
7	6,77288669	0,56074271	0,07856699	2,25354196	3,23537448	4,62544866	9,80788785	13,6149787	18,6479626
7,5	6,67563633	0,56315015	0,06487263	2,22916953	3,19067947	4,55606551	9,69132724	13,5035214	18,5860747
8	6,59895632	0,56523243	0,05373838	2,20941334	3,15495373	4,50099803	9,60050019	13,4193623	18,5471398
8,5	6,54487005	0,5668514	0,04583713	2,1948044	3,12918273	4,46177397	9,53723932	13,3624274	18,5255684
9	6,50969373	0,56799493	0,04119335	2,18423156	3,11163898	4,43585945	9,4962496	13,3251279	18,5098687
9,5	6,48712239	0,5687442	0,03935167	2,17588071	3,09935886	4,41882798	9,46924194	13,2975382	18,4889829
10	6,4704409	0,56921615	0,03970123	2,16781652	3,0891689	4,40593609	9,44759943	13,2699498	18,453652
10,5	6,45349546	0,56953636	0,04166231	2,15820447	3,07810841	4,39279978	9,42353056	13,2338363	18,3964092
11	6,43174062	0,56983253	0,04468979	2,14570213	3,06395692	4,37612156	9,39157065	13,1838877	18,3141939
11,5	6,4024091	0,57020654	0,04824213	2,12968223	3,04545652	4,35389804	9,34867707	13,1179727	18,2080493
12	6,36333707	0,57072276	0,05173848	2,10994572	3,02183485	4,32466857	9,29246451	13,0346449	18,0796727
12,5	6,31371413	0,57140943	0,05453061	2,08699336	2,99317473	4,28802828	9,22232271	12,9347714	17,9337884
13	6,25651491	0,5722566	0,05589291	2,0628252	2,96156616	4,24628069	9,14296503	12,8265145	17,7850881
13,5	6,19784509	0,57323973	0,05508015	2,04051151	2,93062482	4,2038904	9,06359865	12,7242438	17,6567616
14	6,14462969	0,5743261	0,05135492	2,02335142	2,90433608	4,16587813	8,99459881	12,6439489	17,5743191
14,5	6,10045704	0,57547522	0,04419618	2,01324801	2,88492359	4,13493795	8,94132486	12,5941922	17,5523436
15	6,06296414	0,57663485	0,03350874	2,00961885	2,87147375	4,10962122	8,90011978	12,5699836	17,585005
15,5	6,02595217	0,57774372	0,0195784	2,01013567	2,86096163	4,08578129	8,86139658	12,5570855	17,6519482
16	5,98369619	0,57873043	0,00283851	2,01242754	2,85046921	4,05956385	8,8160562	12,5414066	17,7320733
16,5	5,93373402	0,57955743	-0,0162164	2,01496039	2,83841784	4,02919927	8,75990342	12,5157192	17,8136011
17	5,87728789	0,58025114	-0,0370504	2,01716231	2,82472047	3,99522725	8,69450105	12,481249	17,8968578
17,5	5,81735663	0,58088387	-0,0590894	2,01894126	2,81000118	3,95927719	8,62429426	12,4435888	17,9885561
18	5,7569314	0,58151963	-0,0817701	2,02037658	2,79500158	3,9230293	8,55370258	12,4082967	18,0952665
